# Renal Cell Carcinoma Metastasis to the Hamate Bone: A Case Report

**DOI:** 10.1155/cro/6146000

**Published:** 2026-06-19

**Authors:** Abdullah Serin, Melih Oral, Mazlum Veysel Sili, Akin Uzumcugil, Baris Kafa, Nursah Tugtekin, Kemal Kosemehmetoglu, Mehmet Ayvaz

**Affiliations:** ^1^ Department of Orthopedics and Traumatology, Hacettepe University, Ankara, Türkiye, hacettepe.edu.tr; ^2^ Idil State Hospital, Idil, Türkiye; ^3^ Division of Hand Surgery, Department of Orthopedics and Traumatology, Hacettepe University, Ankara, Türkiye, hacettepe.edu.tr; ^4^ Department of Pathology, Hacettepe University, Ankara, Türkiye, hacettepe.edu.tr

**Keywords:** curettage and cementation, hamate bone, internal fixation, osteolytic lesion, renal cell carcinoma

## Abstract

Renal cell carcinoma (RCC) rarely metastasizes to the carpal bones. We describe a 43‐year‐old woman with a history of RCC who presented with ulnar‐sided wrist pain. Imaging demonstrated complete osteolytic destruction of the hamate. Treatment consisted of excision of the hamate, intralesional curettage, cement reconstruction, and internal fixation to the adjacent carpal bones and the fourth and fifth metacarpals. At 15 months of follow‐up, no local recurrence, carpal instability, or implant‐related complications were observed. Surgical excision with cementation and internal fixation achieved local tumor control, maintained carpal stability, and enabled early functional recovery in this rare case of hamate metastasis from RCC.

## 1. Introduction

Renal cell carcinoma (RCC) originates from renal tubules and is the most common type of renal cancer [1]. RCC remains associated with poor long‐term survival, with reported 5‐year survival rates below 10% in advanced disease. Surgical treatment has been shown to improve disease control and patient quality of life [2, 3]. In patients with metastatic RCC, skeletal metastases are observed in roughly 20% of cases and are associated with considerable morbidity as well as impaired quality of life [2, 4]. Early recognition and evaluation have provided a better quality of life and prevented complications [2]. Although there is much variability in the way that RCC is treated, it is now believed that metastasectomy plays a crucial role in patient survival [5]. RCC metastases to the bones of the hand are infrequent, and carpal bone metastases are extremely rare [6, 7]. We report the case of a 43‐year‐old patient with an RCC metastasis to the hamate bone, treated with intralesional curettage, cement augmentation, and internal fixation using two plates, with 15 months of clinical and radiographic follow‐up.

## 2. Case Presentation

A 43‐year‐old right‐hand‐dominant female was referred from the medical oncology department and assessed in our outpatient clinic for pain in her left wrist. Her medical history was notable for epilepsy since the age of 5 and hypertension for the last 7 years. In 2019, she underwent nephrectomy for left RCC staged as T3aN0M0.

Thereafter, in 2021, she manifested pulmonary metastases and underwent stereotactic body radiation therapy (SBRT) at a dose of 50 Gy in five fractions.

**Figure 1:**
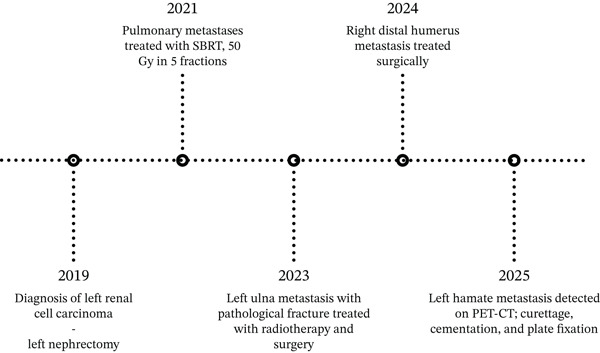
Timeline of the patient′s diagnosis with the relevant data about the treatment.

In 2023, a metastatic lesion developed in the left ulna and was treated with palliative radiotherapy, followed by surgical intervention due to a pathological fracture at an outside institution. Subsequently, a metastatic lesion in the right distal humerus was treated surgically at our center in 2024. Systemic therapy was initiated following the development of metastatic disease, and the patient was later treated with immunotherapy (nivolumab). The carpal lesion was detected during routine positron emission tomography–computed tomography (PET‐CT) follow‐up in 2025. At that time, no additional new metastatic sites were identified, and the disease was considered clinically stable. The chronological sequence of disease progression is summarized in Figure 1. During routine follow‐up with PET‐CT, increased radiotracer uptake was observed in the hamate bone of the left wrist, necessitating referral to our department for further evaluation. The patient suffered from ulnar‐sided wrist pain for the past 2 months, which was elicited by palpation and aggravated during wrist range‐of‐motion assessment. On physical examination, there was localized tenderness over the ulnar aspect of the wrist, with pain exacerbated by ulnar deviation and gripping activities.

**Figure 2:**
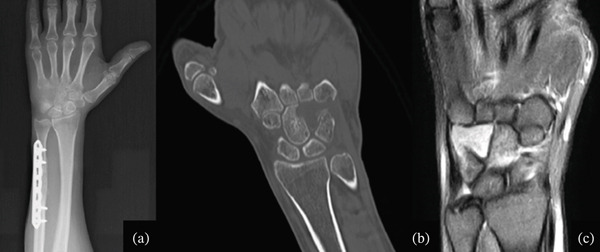
(a) Preoperative anteroposterior radiograph showing a destructive hamate lesion. (b) Computed tomography scan reveals cortical destruction of the hamate and minimal involvement of the capitate. (c) T2‐weighted STIR coronal magnetic resonance image demonstrating a hyperintense signal within the lesion.

Radiography revealed osteolytic destruction of the entire hamate bone (Figure 2a).

A follow‐up CT scan of the wrist revealed complete loss of the hamate (Figure 2b). Magnetic resonance imaging revealed tumor infiltration into surrounding soft tissue and associated edema (Figure 2c). The laboratory results were within normal limits. The Eastern Cooperative Oncology Group performance status was classified as Grade 1. Subsequent to the diagnosis of RCC and accompanying surgical intervention, the patient resigned from her job and has since adopted the responsibilities of a homemaker.

Surgical management consisting of curettage, cementation, and plate fixation was planned.

A volar approach to the left wrist was performed through the ulnar aspect, and Guyon′s canal was opened to expose the palmar surface of the hamate. The ulnar neurovascular bundle was identified and carefully released. A separate dorsal approach was subsequently performed. The dorsal cutaneous branch of the ulnar nerve was identified and protected, and the extensor tendons were retracted. Intraoperatively, the hamate was found to be completely infiltrated by metastatic disease and was therefore excised. A portion of the metastatic capitate was curetted. Following excision, the resulting defect and the curetted area of the capitate were filled with bone cement. Polymethylmethacrylate (PMMA) bone cement was used for reconstruction. The fixation construct was achieved by anchoring the plates and screws to the adjacent intact carpal bones and the fourth and fifth metacarpals, rather than relying on fixation within the cement itself.

**Figure 3:**
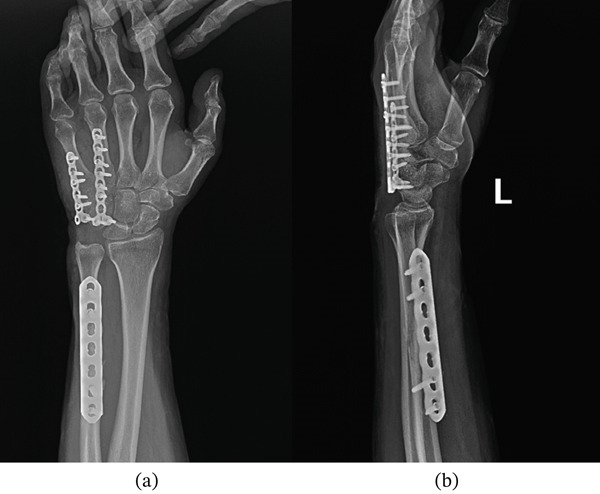
(a) Postoperative anteroposterior radiograph. (b) Postoperative lateral radiograph.

**Figure 4:**
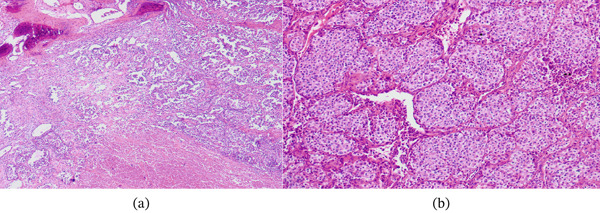
(a) Neoplastic infiltration of lamellar bone (upper left) with accompanying necrosis (lower) (H&E, ×40). (b) Tumor composed of pleomorphic polygonal cells arranged in a nested architecture separated by fibrous septa, with clear to eosinophilic cytoplasm, irregular nuclei, prominent nucleoli (ISUP Grade 3) (∗), and central necrosis (∗∗) (H&E, ×100).

The construct was stabilized by fixation to the adjacent carpal bones as well as the fourth and fifth metacarpals (Figure 3). The excised specimen was sent for histopathological examination, which was consistent with metastatic RCC (Figure 4). No additional local adjuvant treatment (such as phenolization or cryotherapy) was used apart from intralesional curettage. The early postoperative course was uneventful, with no wound complications, neurovascular deficits, or signs of infection.

In November 2025, 15 months later, a biopsy conducted due to the growth of a left axillary lymph node indicated findings consistent with metastatic cancer. The patient is presently undergoing nivolumab treatment. The patient is followed jointly by the medical oncology and orthopedic surgery departments. Surveillance is conducted every 3 months with whole‐body PET‐CT and clinical examination to monitor for both systemic disease progression and local recurrence at the operative site. At the latest follow‐up, 15 months postsurgery, there were no clinical or radiological indications of hamate metastatic recurrence or wrist instability.

The patient exhibited the following active range of motion: wrist extension/flexion 30‐0‐0° and radial/ulnar deviation 5‐0‐0°. The patient was capable of utilizing her hand without pain for daily activities. Prior to the onset of wrist symptoms, the patient reported no functional complaints involving the left wrist and was able to perform all daily activities without limitation; however, a formal preoperative functional assessment such as the QuickDASH score was not obtained. The final follow‐up QuickDASH score was 35, signifying a significant degree of impairment. Follow‐up radiographs demonstrated stable cement reconstruction and plate fixation without evidence of loosening, carpal instability, or local recurrence. The patient was pleased with the surgery outcomes. Despite the moderate functional limitation reflected by the QuickDASH score, the patient reported effective pain relief and expressed satisfaction with the surgical outcome in terms of daily activities.

## 3. Discussion

Bone metastases from RCC are common and contribute to a worsened quality of life in patients due to excessive discomfort and pathological fractures [8]. A solitary bone metastasis is a suitable candidate for extensive surgical excision to improve survival [9]. Acrometastases are typically associated with advanced systemic disease and have been reported to carry a poorer prognosis compared with other solitary metastases.

To the best of our knowledge, this is the first reported case of RCC metastasis to the hamate bone, although isolated metastases to other carpal bones, including the trapezium, have been previously described [10, 11]. A comprehensive literature search was conducted using the PubMed, Scopus, and Web of Science databases using the keywords “renal cell carcinoma,” “hamate,” “carpal metastasis,” and “acrometastasis.” Articles published between January 2000 and May 2026 were screened. Case reports and case series reporting metastatic involvement of the hamate or carpal bones from RCC were included. Review articles and animal studies were excluded, and no previously reported cases of RCC metastasis to the hamate bone were identified.

Nakagawa et al. [11] published a case of trapezium metastasis managed with excision and repair using a nonvascularized iliac bone graft, with no signs of recurrence observed at 2.5 years of follow‐up. Leaving the defect unreconstructed following the resection of a carpal metastasis may increase the risk of carpal instability, especially in patients with prolonged survival; accordingly, addressing the defect seems to be a more logical strategy to preserve carpal stability. To our knowledge, there are no reported studies describing the surgical therapy using cementation for carpal metastases arising from RCC. Cementation was favored not only to restore carpal stability but also to offer rapid mechanical support, promote early mobilization, mitigate graft‐related morbidity, and potentially improve local tumor management.

In the present case, the surgical approach was primarily palliative due to the presence of multiple metastatic sites, including the lung, ulna, and humerus. Therefore, wide excision or amputation was not considered appropriate. Compared with biological reconstruction methods such as bone grafting, cement augmentation offers immediate mechanical stability, avoids donor‐site morbidity, and allows early mobilization.

The main limitations of this report are its single‐case design and the lack of a preoperative functional assessment for objective baseline comparison.

Surgical resection followed by cementation and fixation resulted in early pain relief along with favorable functional and radiological outcomes, supporting cementation as a viable option for carpal metastases of RCC.

## Funding

No funding was received for this manuscript.

## Disclosure

All authors have read and approved the final submitted manuscript.

## Consent

The patient was informed that her clinical data would be submitted for publication, and written consent was obtained from the patient.

## Conflicts of Interest

The authors declare no conflicts of interest.

## Data Availability

The data that support the findings of this study are available from the corresponding author upon reasonable request.
